# Implementation of Lightweight Convolutional Neural Networks with an Early Exit Mechanism Utilizing 40 nm CMOS Process for Fire Detection in Unmanned Aerial Vehicles

**DOI:** 10.3390/s24072265

**Published:** 2024-04-02

**Authors:** Yu-Pei Liang, Chen-Ming Chang, Ching-Che Chung

**Affiliations:** Department of Computer Science and Information Engineering, Advanced Institute of Manufacturing with High-Tech Innovations, National Chung Cheng University, Chia-Yi 621301, Taiwan; ypliang@cs.ccu.edu.tw (Y.-P.L.);

**Keywords:** unmanned aerial vehicles (UAVs), fire detection, neural networks, quantization, fixed-point arithmetic, real-time systems, early-exit mechanism, digital circuits

## Abstract

The advancement of unmanned aerial vehicles (UAVs) enables early detection of numerous disasters. Efforts have been made to automate the monitoring of data from UAVs, with machine learning methods recently attracting significant interest. These solutions often face challenges with high computational costs and energy usage. Conventionally, data from UAVs are processed using cloud computing, where they are sent to the cloud for analysis. However, this method might not meet the real-time needs of disaster relief scenarios. In contrast, edge computing provides real-time processing at the site but still struggles with computational and energy efficiency issues. To overcome these obstacles and enhance resource utilization, this paper presents a convolutional neural network (CNN) model with an early exit mechanism designed for fire detection in UAVs. This model is implemented using TSMC 40 nm CMOS technology, which aids in hardware acceleration. Notably, the neural network has a modest parameter count of 11.2 k. In the hardware computation part, the CNN circuit completes fire detection in approximately 230,000 cycles. Power-gating techniques are also used to turn off inactive memory, contributing to reduced power consumption. The experimental results show that this neural network reaches a maximum accuracy of 81.49% in the hardware implementation stage. After automatic layout and routing, the CNN hardware accelerator can operate at 300 MHz, consuming 117 mW of power.

## 1. Introduction

In the past few years, the use of unmanned aerial vehicles (UAVs) has become increasingly important in the realm of edge computing. These UAVs, noted for their small size and light build, can effectively perform a wide range of tasks across different climates and landscapes. This versatility has led to their widespread use in fields like agriculture monitoring, traffic control, environmental observation, and search and rescue missions. Traditionally, in cloud computing, collecting data involves sending them from edge devices to the cloud, where they are then computed and processed. This method, however, faces several challenges such as network delays, concerns over data security, and privacy issues. UAVs offer a solution by facilitating computation and processing directly on the device itself, which has spurred the development of many new technological applications.

Drones, also known as UAVs, have become increasingly prominent in environmental monitoring, with wildfire detection emerging as a particularly noteworthy application due to the critical need for early fire identification. An example is found in [1], which introduces a method for the early detection of forest fires. Wildfires cause extensive ecological harm to natural habitats like forests, farmlands, and various ecosystems, occurring with disturbing regularity each year. The authors of [1] have developed an algorithm that detects and tracks forest fires by analyzing the color characteristics of flames, allowing for the identification and monitoring of fire-affected areas. Contrastingly, ref. [2] introduces a different approach utilizing environmental sensors. This technique involves monitoring elements such as temperature, carbon monoxide (CO), and carbon dioxide (CO_2_) levels to detect early signs of fire. In a similar vein, ref. [3] proposes a method that focuses on smoke detection as an indicator of fire conditions.

**Figure 1:**
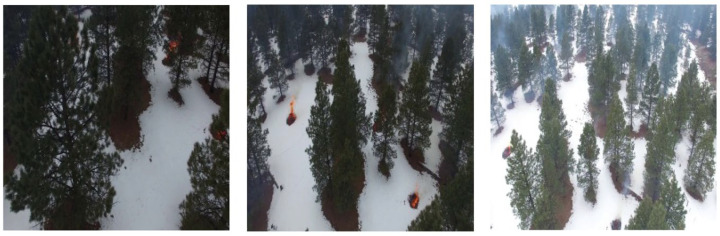
Sample images of pile burn.

In forest management, controlled burns are routinely conducted to clear debris like twigs and leaves. Traditionally, this process of controlled burning demands extensive monitoring over several days by staff to prevent the fire from becoming unmanageable. Fortunately, with the technology of automated aerial surveillance systems of UAVs, real-time images can be captured easily and further processed automatically to detect wildfires in time. For example, Alireza [4] introduces a FLAME dataset specifically gathered from UAVs during intense fire events. The authors utilize Google’s Xception model [5] for categorizing fires, achieving accuracy rates of 96.79%, 94.31%, and 76.23% in the training, validation, and testing phases, respectively. Moreover, the dataset they used has been published in IEEE Data Port. Specifically, this dataset was collected in northern Arizona and includes a comprehensive array of images and videos of fires. Figure 1 shows sample images of pile burns from the dataset, captured from various perspectives with different zoom levels and camera types. Such datasets focusing on intense fires are crucial for analyzing the initial phases of fire incidents.

As mentioned above, the potential for automatically detecting wildfires using UAVs has been explored. The process involves UAVs cruising in the air above forests, capturing real-time images periodically. Subsequently, the onboard processors on the UAVs can promptly process these images to detect wildfires and trigger alarms if any unusual situations are detected. This proactive approach to wildfire detection holds promise for enhancing early warning systems and mitigating the risk of forest fires.

Therefore, Rafik [6] introduces a novel classification framework tailored for the previously mentioned fire detection dataset. This framework integrates EfficientNet-B5 [7] with DenseNet-201 [8], employing them for feature extraction in wildfire classification. This combined architecture reports an accuracy of 85.12%. However, considering the parameters of EfficientNet-B5 and DenseNet, this model’s practicality for UAV deployment warrants further consideration. In a related development, Lin [9] presents a transfer learning-based model, FT-ResNet50. This model adapts a ResNet network, originally trained on the ImageNet dataset, to the context of forest fire detection. The authors refine ResNet using the Adam optimization algorithm and the Mish activation function. Additionally, they enhance its performance with the Focal loss function, achieving an accuracy of 79.48%. While the integration of transfer learning and fine-tuning improves image classification, an accuracy of 79.48% may not be sufficient for precise image categorization.

Integrating AI technology into fire detection processes can streamline operations and improve the efficiency of drones in executing their tasks in the aforementioned studies. However, the computational requirements of deep neural networks often surpass the processing capabilities available on drone. On the other hand, recently, numerous solutions have emerged for accelerating machine-learning-based computations, including commercial-off-the-shelf products like Jetson Nano boards for convolutional neural network (CNN), field-programmable gate array (FPGA) custom accelerators, coarse-grained reconfigurable array (CGRA) approaches [10], and automatically generated AI hardware accelerators [11]. However, these solutions often overlook the power consumption issue. In summary, though there have been many excellent related works and technologies in the past, there has not been an integrated solution that can simultaneously meet the requirements of low energy consumption, high efficiency, and high accuracy.

Given our focus on wildfire detection technology using UAVs, power consumption is a crucial consideration. In this paper, we propose an ASIC-based solution to mitigate power consumption concerns in UAVs. Specifically, we developed a streamlined CNN model tailored for wildfire detection, leveraging a dataset collected via drones. This model incorporates several early exit points to alleviate computational burden and reduce energy consumption. Furthermore, the inclusion of multiple early exit points enhances flexibility by offering users various usage modes to choose from. Additionally, quantization methods were applied to both the weights and activations to optimize storage demands. Moreover, a specialized hardware circuit was implemented using TSMC 40 nm CMOS technology, and its power consumption was assessed during circuit operations.

The contributions of this study can be summarized as follows:

1. Development of a lightweight CNN model for wildfire recognition, incorporating multiple exit points;
2. The proposed model substantially reduces neural network computations while maintaining an 83% accuracy rate in predictions;
3. Significant reduction in the memory requirements and energy consumption of the hardware circuit, with only a slight decrease in CNN accuracy;
4. Implementation of the hardware circuit using TSMC 40 nm CMOS technology, enhanced with power gating techniques to further reduce power consumption;
5. The implemented ASIC offers multiple usage modes for users to select according to various usage scenarios.

The structure of the remaining paper is organized as follows: Section 2 delves into various model compression techniques, focusing on adapting machine learning models for edge computing. Section 3 and Section 4 introduce the proposed method. Specifically, Section 3 outlines the software architecture of our proposed model, while Section 4 details the hardware implementation. The experimental results are presented in Section 5. Finally, Section 6 concludes this work.

## 2. Related Work

Deploying deep neural networks on edge devices is challenging due to their substantial size. As a result, a variety of model compression techniques have been developed. Cheng [12] identifies key methods for reducing model size, including network pruning, weight sharing, knowledge distillation, and early model termination. Network pruning [13] is a strategy aimed at reducing the size of a neural network by removing superfluous neurons and weights. This involves eliminating unnecessary parts of a pre-trained model and retraining the remaining sections to ensure the model’s performance is largely maintained.

Weight sharing is a neural network model compression technique aimed at reducing the number of weights and minimizing storage needs. Its core principle involves grouping similar weight values within the neural network into clusters and representing each weight by the centroid value of its cluster. This ensures that weights with close values share the same representative value. Such a strategy reduces the model’s storage space requirements and can decrease computational load without significantly impacting performance. Deep compression [14] is introduced as a three-step model compression process: pruning, quantization, and Huffman coding. Pruning involves eliminating weights below a certain threshold, followed by retraining the network with its newly sparse connections until it converges. K-means clustering is then used to allow weights in the same cluster to share a common centroid, with fixed-point processing applied. Weight updates involve accumulating gradients within each cluster, adjusting them with a learning rate, and subtracting from the shared centroid to refine the centroids. Finally, Huffman coding is employed to compress weights and indices further.

Knowledge distillation [15] is a technique for model compression that involves transferring insights from a larger, more complex model to a smaller, simpler one. This allows the smaller model to leverage the advanced understanding of the larger model, potentially improving its performance. On the other hand, early model termination [16] focuses on embedding early exit points within the original model. These exits allow the model to halt computations prematurely when it has high confidence in its prediction, thereby saving computational resources and time. The most basic form of early exit relies on a confidence threshold. If the classifier’s output confidence surpasses this threshold, the model ceases operation and outputs the prediction. However, if the confidence is below the threshold, the model continues to the final classifier for a conclusive prediction result.

Conversely, a binary neural network (BNN) [17] is a form of deep neural network that utilizes binary values for both weights and activations. This method quantizes the original network’s weights and activations to 1 bit, effectively converting 32-bit floating-point numbers (FP32) into either +1 or −1. Although this significantly reduces the model’s storage requirements and enhances inference speed, it comes with a notable trade-off of considerable information loss, leading to a reduction in accuracy. In comparison, ternary weight networks (TWN) [18] add an extra value of 0 to the binary scheme of +1 and −1 for weight conversion. TWN occupies 2 bits per weight, enabling a 16-fold decrease in storage needs compared to standard 32-bit floating-point formats. TWN typically achieves higher accuracy than BNN. Research shows that TWN’s quantization method can effectively compress several well-known models.

Furthermore, mixed precision training [19] employs a combination of 16-bit floating-point numbers (FP16) and FP32 for storing weights, activations, and gradients during the training of neural networks. This approach reduces both memory usage and computational complexity, resulting in faster training speeds while maintaining accuracy. In addition, DoReFaNet [20] utilizes an asymmetric quantization strategy, allowing for the quantization of weights, activations, and gradients to any specified bit-width. This increases the flexibility of quantization. The quantization process, represented as quantize(k), is described in Equation (1). This equation quantizes an input, x, into k bits. Equation (2) illustrates the method for quantizing low-bit weights, where the quantized weights are confined to k-bit fixed-point numbers ranging from −1 to 1.

(1) $${quantize}_{k}\left(x\right)=\frac{1}{2^{k}-1}round\left(\left(2^{k}-1\right)x\right)$$

(2) $$f_{w}^{k}\left(r\right)=2\times {quantize}_{k}\left(\frac{\tanh\left(r\right)}{2max(|\tanh\left(r\right)|)}+\frac{1}{2}\right)-1$$

## 3. The Proposed Hardware Architecture

### 3.1. CNN Architecture Overview

**Figure 2:**
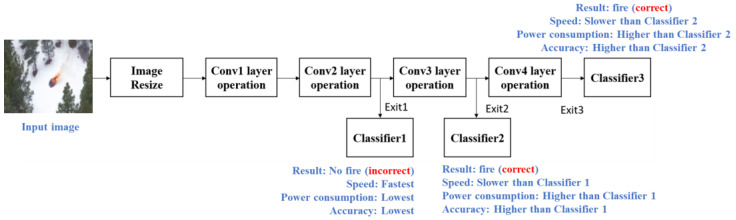
CNN model architecture with early exit mechanism.

To balance the accuracy and efficiency, this paper presents a CNN equipped with an early exit mechanism specifically tailored for fire detection using UAVs. Note that the FLAME dataset [4] is utilized in this paper, comprising 25,018 images featuring fire and 14,357 images without fire for training. Furthermore, 20% of the data are designated for validation, while the test set includes 5137 images with fire and 3480 without. The proposed model architecture with an example using a scenario is depicted in Figure 2. As shown in Figure 2, the proposed model features two additional early exit points in addition to the standard final exit point. This design is optimized for UAV fire detection and offers the flexibility to switch between exit points based on various factors, such as application scenarios, the complexity of the classification task, and power-saving requirements. More specifically, as depicted in Figure 2, among the multiple exit points, the inference speed is maximized and power consumption minimized at the earliest exit. However, the accuracy is comparatively lower than that of later exit points. Therefore, our proposed solution offers various operational modes tailored to different circumstances. For instance, we may initially designate exit 1 as the default mode to swiftly acquire results and conserve power. Upon detecting unusual conditions, the controller transitions to a higher accuracy mode (later exit points) to verify the situation. Alternatively, exit 3 can be employed as the default mode for optimal accuracy. In instances of low battery, the mode can then be shifted to the early exit points to conserve power.

**Figure 3:**
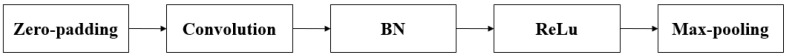
The complete operation of each layer.

The convolutional operations within the model, as illustrated in Figure 3, follow a series of calculated steps to improve feature extraction. It begins with zero padding, which helps to retain essential image information. The convolutional stride is set to 1, effectively expanding the model’s receptive field and allowing it to cover a broader contextual area. Following convolution, batch normalization (BN) is integrated, accelerating the model’s optimization process and providing regularization advantages. At the end of the convolutional sequence is a pooling layer. Here, a pooling stride of 4 is deliberately chosen to decrease the dimensions of the resulting feature maps. This reduction is critical for the model’s subsequent hardware implementation, as it considerably reduces the computational load.

**Figure 4:**
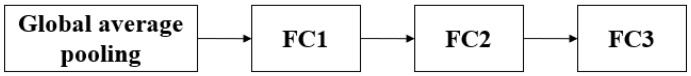
The complete operation of each classifier.

After the convolutional operations are complete, the classifier’s computations begin, as illustrated in Figure 4. The process starts with global average pooling applied to the feature map, which not only reduces the number of model parameters but also enhances the model’s ability to generalize. Following this step, the processed data are fed into fully connected layers for image classification. Importantly, dropout layers are inserted between the three consecutive fully connected layers. Each of these layers has a dropout rate of 50%, acting as a preventive measure against model overfitting.

Table 1 displays the feature map sizes at each layer of the model. The initial input images are 64 × 64 in dimension. After passing through the pooling layers, the feature maps experience a considerable reduction in size, which is beneficial for the later stages of hardware implementation.

Table 2 thoroughly details the parameterization of the entire neural network architecture. The parameters for both the convolutional layers and the batch normalization processes are precisely calculated using the methodologies described in Equations (3) and (4), respectively. Furthermore, the parameters for the fully connected layers are defined according to Equation (5). It is important to note that a significant decrease in the number of model parameters is achieved after implementing global average pooling. By summing up all the individual parameters, the total parameter count of the proposed model is obtained.

(3) Conv parameters=in_channel×kernel size×out_channel

(4) BN parameters=4×kernel size×out_channel

(5) FC parameters=feature map size×out_channel

Moreover, at the specific stages of Exit1, Exit2, and Exit3, the cumulative parameter counts are approximately 2 K, 4.6 K, and 11.2 K, respectively. This detailed breakdown of parameters effectively highlights the complexity and capacity of the model across its various architectural segments.

**Figure 5:**
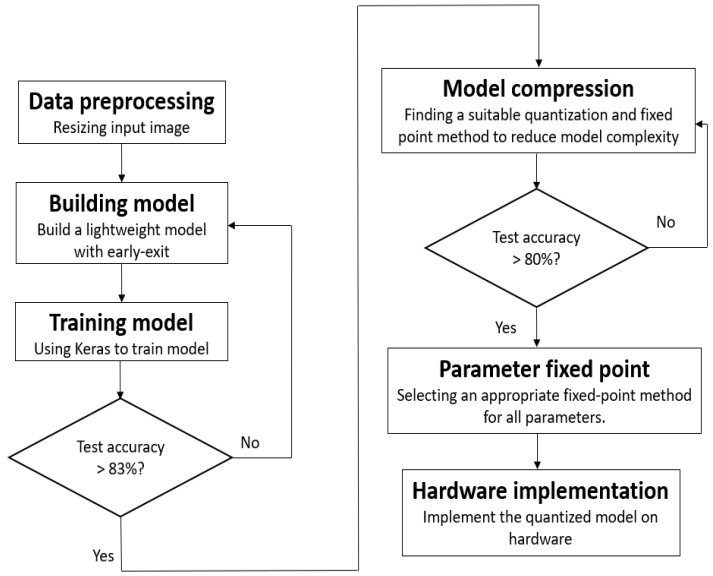
Flow chart for creating a lightweight model with early exit.

The process of architectural construction, depicted in Figure 5, begins with applying suitable preprocessing to the input images. The careful choice of preprocessing techniques significantly influences the subsequent steps. In the field of deep learning, image resizing is commonly achieved through various interpolation methods. These include nearest-neighbor interpolation, bilinear interpolation, and bicubic interpolation, which are among the most frequently used techniques.

Table 3 provides a detailed analysis of how various interpolation techniques affect the accuracy of the proposed architecture. The performance of the framework is assessed using different compression sizes, employing the three interpolation methods mentioned earlier. The results in Table 3 highlight the significant impact of nearest-neighbor interpolation on accuracy, particularly noting a marked decrease in accuracy when images are reduced to 64 × 64 dimensions. In contrast, bilinear and bicubic interpolation techniques yield more favorable outcomes. Considering computational complexity, the architecture ultimately utilizes bilinear interpolation, reducing images to 64 × 64 dimensions.

This thorough analysis not only highlights the complex interplay between interpolation methods and model performance but also justifies the choice of bilinear interpolation in the proposed architecture.

After completing the preprocessing of the dataset, the next step involves selecting a suitable network architecture. Table 4 provides a comprehensive comparison of different choices regarding the number of channels in the convolutional layers within the proposed architecture. The channel count in the initial convolutional layer is of particular importance for overall accuracy. Therefore, Table 4 primarily investigates how varying channel counts in the first convolutional layer influence the model’s total parameter count and accuracy. Insights gleaned from Table 4 reveal that a channel count of four in the first layer yields unsatisfactory accuracy. Conversely, increasing the channel count to eight leads to a significant improvement in accuracy. Further increases to 16 and 32 channels result in only marginal enhancements. Taking into account computational complexity, the architecture is ultimately designed with eight channels in the first convolutional layer.

After establishing the model’s depth, the proposed architecture incorporates an early exit mechanism to reduce computational costs for edge device deployment. Table 5 offers a comprehensive analysis of the inference accuracy at different exit points within the architecture. The findings reveal that Exit1 exhibits relatively lower accuracy, whereas the distinctions in accuracy between Exit2 and Exit3 are minimal. This thorough examination not only underscores the critical significance of the channel count in the initial convolutional layer for accuracy but also underscores the efficacy of the early exit strategy. Such an approach proves especially advantageous for optimizing computational resource utilization during inference, particularly in edge computing environments.

After completing the model’s construction, the next essential step is its quantization. Due to the limited computational resources available on edge devices, it is necessary to reduce the model’s computational demands and minimize storage space usage. However, it is crucial to acknowledge that quantization often leads to a decrease in model accuracy. If this reduction in accuracy is significant, it could undermine the model’s practicality. Therefore, the primary challenge lies in achieving an optimal balance between maintaining model accuracy and reducing computational requirements, a factor of paramount importance.

### 3.2. Weight Quantization Method

In the inference phase of neural networks, using floating-point numbers for weight storage can lead to significant storage demands. Therefore, when performing inference computations on edge devices, it becomes crucial to quantize these floating-point numbers from their standard 32-bit format to a lower-bit precision. In the proposed architecture, the quantization approach is based on DoReFaNet [20], as outlined in Section 2. DoReFaNet introduces a quantization function that converts continuous weight values into a discrete range between −1 and 1, as shown in Equation (2). This section focuses on determining the appropriate quantization bit precision, aiming to strike a balance between maintaining weight accuracy and reducing storage needs.

Table 6 clearly illustrates the impact of applying different quantization bit precisions on the accuracy at each exit point within the same model. It is observed that when weights are quantized to 7 bits, there is only a slight decrease in accuracy at all three exit points. In contrast, quantizing the weights to 6 bits results in a more significant reduction in accuracy. Consequently, the decision is made to quantize the weights at 7 bits. This choice effectively balances the need to maintain accuracy with the goal of reducing storage requirements.

### 3.3. Software Results

After applying DoReFaNet’s quantization technique via the software platform, initial results from the proposed network architecture have emerged. Table 7 displays the confusion matrix for this architecture. Following detailed calculations, the performance metrics obtained on the FLAME dataset are as follows: the model achieved an accuracy of 82.69%, a precision of 83.38%, a recall of 87.04%, and an F1 score of 85.17%.

## 4. Hardware Implementation

### 4.1. Fixed-Point of Activation Values and Parameters

Writing feature maps back to memory is crucial to implementing a convolutional neural network. Therefore, reducing the bit-width for activations becomes important during hardware computations. Experiments within the proposed architecture have shown that using fixed-point quantization for activations does not markedly affect accuracy.

Table 8 shows the accuracy levels associated with different fractional bit-widths for activations. It is apparent that maintaining a fractional bit-width of 5 results in only minor accuracy changes. In contrast, reducing the bit-width to 4 leads to a significant decrease in accuracy. Therefore, the ideal setting involves using a fractional bit-width of 5, which requires only a quarter of the memory needed for storing activations in the original FP32 format. Furthermore, minimizing the bit-width of activations saves memory and reduces the computational time for processing these values. Such optimization enables convolutional neural networks to perform more efficient inference and faster execution, making them more suitable for resource-limited settings and real-time applications.

After the model training is finalized, the four parameters of the batch normalization (BN) layer become fixed. These BN parameters undergo quantization to reduce memory consumption for future hardware implementations. Prior to quantization, the four BN parameters are condensed into two, as described in Equations (6) and (7). These two parameters are $\mu_{\beta }$, which represents the mini-batch mean, and $\alpha_{\beta }^{2}$, representing the mini-batch variance.

After simplifying the parameters, the total count of parameters in batch normalization is reduced by half, from 128 to 64. This reduction simplifies the numerical calculations to just one multiplication and one addition, as illustrated in Equation (8). Such simplification is beneficial for the upcoming hardware implementation. Table 9 also explores the effects of separately quantizing the variables $\gamma^{\prime }$ and $\beta^{\prime }$, with a particular focus on the resultant error. Initially, the value of $\beta^{\prime }$ is held constant while adjusting the bit-width for quantizing $\gamma^{\prime }$. Table 9 shows the accuracy outcomes following the quantization of $\gamma^{\prime }$.

(6) $$\gamma^{\prime }=\frac{1}{\sqrt{\alpha_{\beta }^{2}}}\gamma$$

(7) $$\beta^{\prime }=-\left(\mu_{\beta }\gamma^{\prime }\right)+\beta$$

(8) $$y_{i}=\gamma^{\prime }x_{i}+\beta^{\prime }$$

Table 9 reveals that maintaining a fractional bit width of 8 or more results in relatively stable accuracy at all three exit points. However, there is a noticeable decrease in accuracy when the fractional bit width is reduced to 7. Therefore, for quantizing the variable $\gamma^{\prime }$, a fractional bit width of 8 is ultimately selected.

Table 10 illustrates the effects of quantizing $\beta^{\prime }$ at various bit widths on model accuracy. The data shows that when the fractional bit width is set above 4, the accuracy decrease is minimal. However, reducing the fractional bit width to 3 results in a substantial decline in accuracy, particularly at Exit2 and Exit3.

The batch normalization parameters will be implemented using lookup tables during the hardware implementation phase. Additionally, as discussed in Section 2, the quantized weights from DoReFaNet will also be stored in lookup tables. These weights will be treated as fixed-point numbers. Since DoReFaNet limits the weight range to −1 and 1, only 2 bits are required to store the integer part of the weights. The next crucial step is deciding the suitable number of fractional bits for storing weights in the lookup table. Table 11 shows a significant drop in overall accuracy occurs when the fractional bit-width is less than 8 bits. Therefore, choosing 8 bits for the fractional part of the weight is optimal, ensuring accuracy is maintained during the storage process in the lookup table.

### 4.2. CNN Hardware Accelerator Architecture

**Figure 6:**
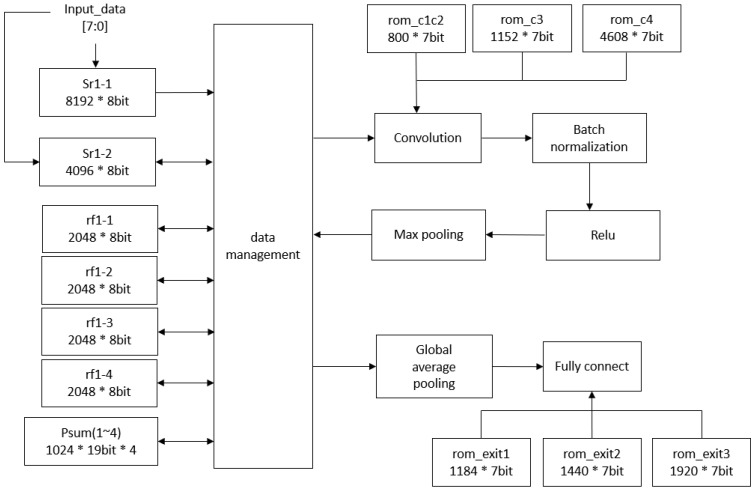
The proposed CNN hardware architecture with early exits.

Figure 6 illustrates the detailed hardware architecture, which includes the computational blocks and memory for storing weights and feature maps. The static random-access memory (SRAM) and register files, created by the memory compiler in the TSMC 40 nm process, are single-port memories. They store input images and the feature maps produced after convolutional layer operations. The Psum1 to Psum4, developed using the logic synthesis tool, are register files that hold the partial sums from each channel in the convolutional layer. The read-only memory (ROM) stores the weights for both the convolutional and fully connected layers. Notably, to reduce physical costs and energy consumption, memory blocks are carefully reused during each inference cycle. This involves breaking down the computation process of the proposed model. Specifically, computations for convolutional layers begin in the convolution block and progress through batch normalization, max pooling, and ReLU activation. The resulting feature maps are then saved to the register file or SRAM, which are subsequently reused for each layer’s computations. After completing two, three, or four convolutional layers, predictions are generated through global average pooling and fully connected layers. This breakdown of the computation process enables the design of a dataflow to reuse memory blocks.

Table 12 provides the utilization of each memory block. The input image is 64 × 64 pixels in RGB format, resulting in three distinct channels. Considering that each word is 8 bits, two memory blocks (Sr1-1 and Sr1-2) are required to store the input, occupying a total of 98.306 KB. The combined channels in the first layer are kept in four Psum registers, amounting to 77.824 KB in size. Following max-pooling, the reduced feature maps are stored in four register files, totaling 65.536 KB. Additional computations on these scaled-down feature maps negate the need for extra storage, allowing these memory spaces to be reused for subsequent calculations. The SRAM and register files are powered down when not in use to save energy.

The storage capacity within Psum1 to Psum4 is divided into four distinct register banks. After thorough testing, a 19-bit representation emerged as the best choice to balance accuracy with reduced storage requirements. Each of these banks has a capacity of 1024 × 19 bits. To enhance power efficiency, the design strategically utilizes all storage blocks only during the initial convolutional layer. From the second convolutional layer onwards, just one register bank is used for channel storage, while the other three are efficiently managed using clock-gating techniques. This approach temporarily suspends their operation, contributing to energy savings.

**Figure 7:**
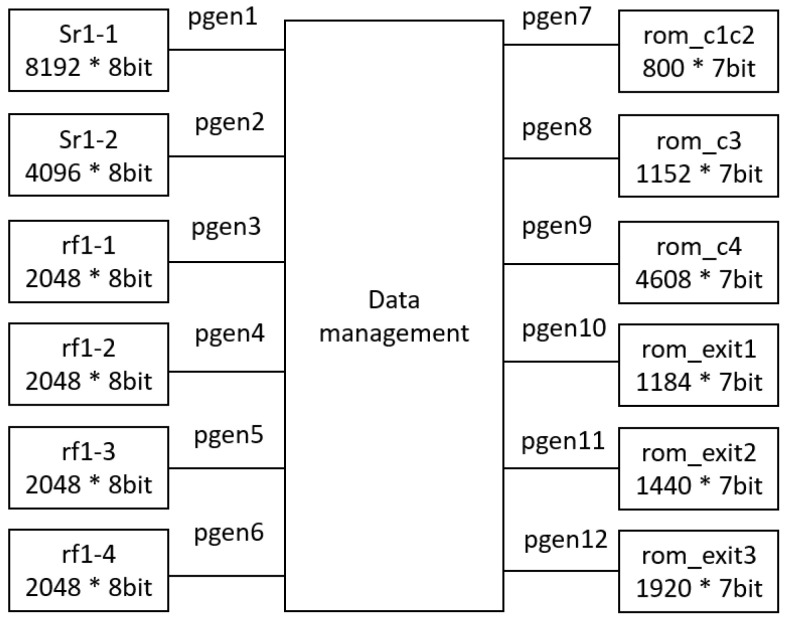
The proposed power gating control.

To mitigate power consumption, a critical concern in UAV applications, the proposed architecture integrates power gating technology. This involves deactivating ROM, RAM, and register components when not in use to conserve power. Therefore, in the hardware design, memory space is segmented, with power supply to segments alternately activated or deactivated based on operational requirements, reducing power usage during memory inactivity. Figure 7 illustrates the power gating control strategy, with control signals pgen1 and pgen2 managing SRAM switches, and pgen3 to pgen6 controlling the register file switches. Additionally, pgen7 to pgen12 regulate ROM switches.

**Figure 8:**
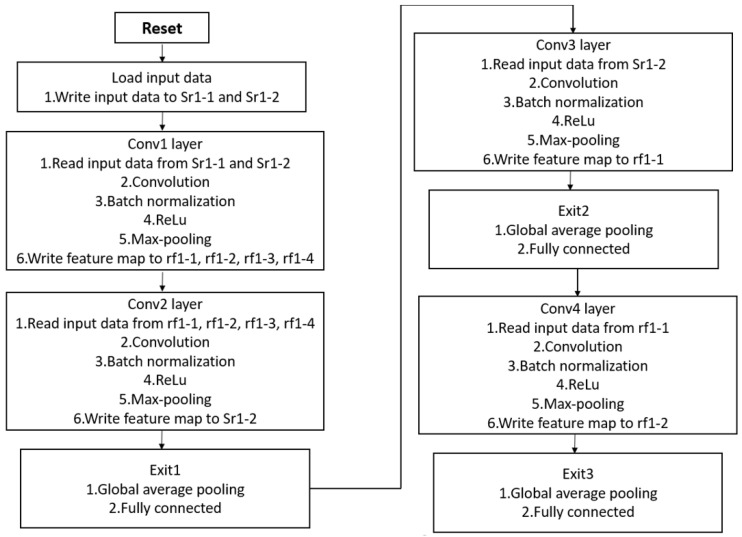
The flow chart for the CNN hardware circuit.

In summary, Figure 8 provides the hardware operation flow for the complete CNN. After processing through the second, third, and fourth convolutional layers, the feature maps proceed to three fully connected layers to generate the output predictions. Additionally, Table 13 shows the significant reduction in memory usage attained through the hardware implementation of the proposed architecture. While the original neural network stored feature maps and weights using FP32, the implementation in this work, involving weight quantization and fixed-point representation for activation values, results in a memory usage reduction of approximately 72%. This substantial decrease is particularly beneficial for edge AI applications, where low computational resource requirements are paramount.

## 5. Experimental Results

The CNN hardware design proposed in this work was implemented using the TSMC 40 nm CMOS process. To evaluate the power consumption, we use an automatic placement and routing tool to collect the experimental data. The design was routed using Cadence Innovus, followed by post-layout simulation to generate the switching activities file. Subsequently, we employed the Powermeter tool of the Cadence Voltus tool, incorporating simulated switching activities and cell library power models, to calculate the power consumption of the design with annotated wire RC effects. During the post-layout simulation phase, the design achieved an operational frequency of 300 MHz. Notably, the proposed CNN hardware accelerator, which incorporates power gating techniques, exhibited a power consumption of only 117 mW at this frequency. The development process involved several critical steps, including quantization and adopting fixed-point representation, which transitioned the model from its design phase to actual hardware implementation. These transformations can potentially alter the model’s accuracy. In the register transfer level (RTL) simulation, intermediate computations were also limited to fixed-point representation, resulting in some errors. Table 14 details the accuracy levels of the proposed architecture at various stages of its development, demonstrating that the architecture, even after undergoing software and hardware implementation phases, maintains an accuracy of over 80% at Exit3.

**Figure 9:**
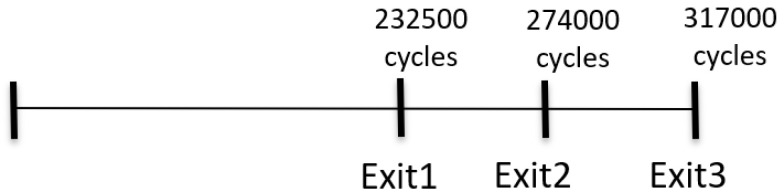
The different situations RTL-level cycles count.

Figure 9 shows the number of cycles needed during the Verilog simulation phase for the proposed architecture to assess the real-time performance. Opting for Exit1 as the prediction point requires 232,500 cycles, while selecting Exit2 takes 274,000 cycles, and Exit3 needs 317,000 cycles. It is evident that choosing Exit1 for the prediction results in a speed approximately 1.4 times faster than using Exit3. This observation is consistent with the anticipated performance of a lightweight model.

**Figure 10:**
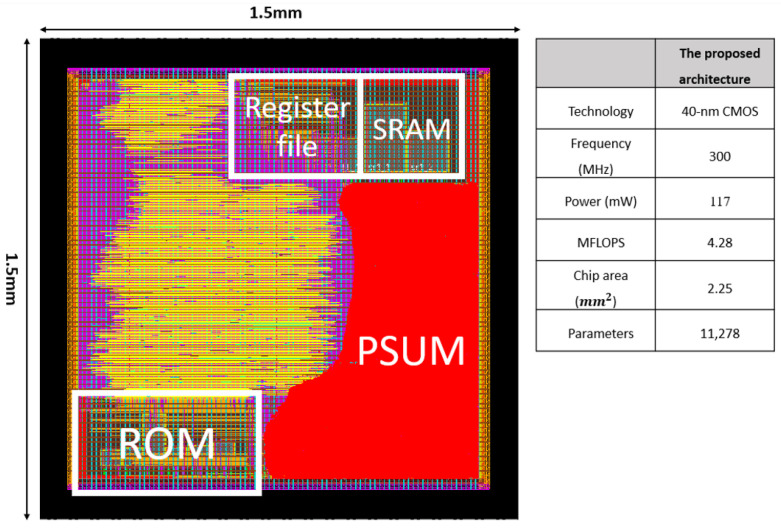
The layout of the proposed CNN hardware circuit.

Figure 10 shows the chip layout of the proposed CNN model, clearly marking the locations of the SRAM, register file, ROM, and Psum components. The total area of the chip is 1500 × 1500 $\mu m^{2}$.

At a working frequency of 300 MHz, the time needed for Exit1 is determined by 232,500 cycles, with each cycle being 3.3 nanoseconds. This results in a total time of 767,250 nanoseconds or approximately 0.077 s. For Exit2, the process involves 274,000 cycles, which total 904,200 nanoseconds or about 0.090 s. Finally, Exit3 necessitates 317,000 cycles, amounting to 1,046,100 nanoseconds, or roughly 0.105 s. Based on these calculations, the proposed architecture is capable of achieving image inference frames per second (FPS) rates of 13.16 for Exit1, 11.11 for Exit2, and 9.615 for Exit3.

To emphasize the effectiveness and simplicity of the proposed CNN model, the architecture was tested on a Raspberry Pi 3 Model B, featuring a 1.2 GHz 64-bit quad-core ARM Cortex-A53 CPU and 1 GB of memory. In this experimental phase, the neural network was refined through weight quantization, as well as fixed-point quantization of activation values and batch normalization (BN) parameters. The inference execution time for a single image was meticulously recorded over several repetitions, revealing an average time of approximately 3.809 s. Thus, it was observed that the Raspberry Pi 3 Model B (Sony UK Technology Centre (UKTec), Pencoed, Wales, UK) has the capability to process about 0.26 images per second in this test setup. However, this rate indicates that relying solely on the Raspberry Pi 3 Model B to run the neural network falls short of meeting the required speed for certain demands.

To highlight the differences between the proposed architecture and prior methods, Table 15 shows a comparative analysis. In the proposed architecture, image resizing to 64 × 64 pixels is implemented as a strategy to minimize network parameters and overall size. The architecture adopts the DoReFaNet quantization technique, which effectively reduces the storage requirements for weights and computation time while still achieving an accuracy of 82.69%. In the hardware implementation phase, a fixed-point methodology is applied to decrease the storage needs for activation values and weights. This hardware is realized using the TSMC 40 nm CMOS process and operates at a frequency of 300 MHz. The results show an FPS range from 9.615 to 13.16, with a maximum power consumption of 117 mW for Exit3. Consequently, the energy per inference for the proposed ASIC can be computed as 317,000 (cycles) × 3.333 × 10^−9^ (s) × 117 × 10^−3^ (W), resulting in 1.23618 × 10^−4^ Joules. This indicates remarkably low energy consumption. On the other hand, in the proposed CNN architecture, the power consumption decreases to 100.12 mW with the selection of Exit1 and 108.59 mW with Exit2. This design shows the architecture’s adaptability, allowing for adjustments to align with specific application scenarios and power-saving needs.

The remaining works [6,8,13] employed computational resources from the NVIDIA RTX GeForce 2080 Ti (NVIDIA Corporation, Santa Clara, CA, USA). However, attaining a precise evaluation of energy consumption during inference necessitates measuring the energy usage of each network using the original hardware and software, which includes employing energy meters. Unfortunately, the energy requirements for inference are rarely reported and the available documentation does not specifically address the power consumption of these resources. According to the manufacturer’s official specifications, the NVIDIA GeForce RTX 2080 Ti typically requires 600 W of power. In contrast, the power consumption of the Raspberry Pi 3 Model B is significantly lower, estimated at around 2.5 W. Therefore, this power information has been included in the table for rough comparison.

## 6. Conclusions

This paper proposes a CNN hardware accelerator featuring early termination options, specifically for identifying wildfires in images taken by UAVs. The structure of this model consists of four convolutional layers, enhanced with batch normalization and max pooling techniques. Each early exit point in the model is equipped with a global average pooling layer, followed by three densely connected layers. During the development of this model, it attained a peak accuracy of 83.11%. The model utilizes the DoReFaNet approach for weight quantization, effectively compressing the weight bits to 7 bits. Remarkably, this CNN model operates with just 11,278 parameters. In the RTL stage, the batch normalization elements are streamlined from four components to two, and a fixed-point numeric format is applied to the weights. At this juncture, the model reached a maximum accuracy of 81.49%. Additionally, the proposed CNN’s hardware framework integrates power gating technology, contributing to reduced energy usage during its implementation phase. This design works at a clock frequency 300 MHz and demonstrates a power consumption of merely 117 mW.

## Figures and Tables

**Table 1:** The input and output data size of each layer.

Operation	Input Data Size (Width × Height × in_channel)	Output Data Size (Width × Height × in_channel)	Stride
Convolution 1	64 × 64 × 3	64 × 64 × 8	1
Max-pooling 1	64 × 64 × 8	32 × 32 × 8	4
Convolution2	32 × 32 × 8	32 × 32 × 8	1
Max-pooling 2	32 × 32 × 8	16 × 16 × 8	4
Global average pooing	16 × 16 × 8	1 × 1 × 8	-
FC 1	1 × 1 × 8	1 × 1 ×30	-
FC 2	1 × 1 × 30	1 × 1 × 30	-
FC 3	1 × 1 × 30	1 × 1 × 1	-
Convolution 3	16 × 16 × 8	8 × 8 × 16	1
Max-pooling 3	8 × 8 × 16	4 × 4 × 16	4
Global average pooing	4 × 4 × 16	1 × 1 × 16	-
FC 4	1 × 1 × 16	1 × 1 × 30	-
FC 5	1 × 1 × 30	1 × 1 × 30	-
FC 6	1 × 1 × 30	1 × 1 × 1	-
Convolution 4	4 × 4 × 16	4 × 4 × 32	1
Max-pooling 4	4 × 4 × 32	2 × 2 × 32	4
Global average pooing	2 × 2 × 32	1 × 1 × 32	-
FC 7	1 × 1 × 32	1 × 1 × 30	-
FC 8	1 × 1 × 30	1 × 1 × 30	-
FC 9	1 × 1 × 30	1 × 1 × 1	-

**Table 2:** The number of parameters of each operation.

Operation	Number of Parameters	Sum of Parameters
Convolution 1	3 × 3 × 3 × 8	216
Batch normalization 1	4 × 8	32
Convolution 2	8 ×3 × 3 × 8	576
Batch normalization 2	4 × 8	32
FC 1	8 × 30	240
FC 2	30 × 30	900
FC 3	30 × 1	30
Convolution 3	8 × 3 × 3 × 16	1152
Batch normalization 3	4 × 16	64
FC 4	16 × 30	480
FC 5	30 × 30	900
FC 6	30 × 1	30
Convolution 4	16 × 3 × 3 × 32	4608
Batch normalization 4	4 × 32	128
FC 7	32 × 30	960
FC 8	30 × 30	900
FC 9	30 × 1	30
Total parameters		11,278

**Table 3:** Comparing the accuracy of various interpolation methods.

Image Size	Nearest Neighbor Interpolation Test Accuracy	Bilinear Interpolation Test Accuracy	Bicubic Interpolation Test Accuracy
128 × 128	74.53%	84.70%	84.64%
64 × 64	65.32%	83.29%	83.11%
32 × 32	54.62%	75.66%	72.42%

**Table 4:** The test accuracy with different channels of convolution layers.

Method	Conv. Layer (in_channel/out_channel)				Sum of Conv. Parameters	Test Accuracy
	Layer1	Layer2	Layer3	Layer4		
1	3/4	4/8	8/16	16/32	6156	73.29%
2	3/8	8/8	8/16	16/32	6552	83.11%
3	3/16	16/8	8/16	16/32	7344	83.57%
4	3/32	32/16	16/16	16/32	12,384	83.64%

**Table 5:** The accuracy of the images at different exit points.

Image Size	Exit1 Accuracy	Exit2 Accuracy	Exit3 Accuracy
64 × 64	79.02%	83.04%	83.11%

**Table 6:** Accuracy of weight quantization with different bits.

Bits	Exit1 Accuracy	Exit2 Accuracy	Exit3 Accuracy
9	78.86%	82.71%	82.81%
8	78.62%	82.57%	82.79%
**7**	**78.35%**	**82.21%**	**82.69%**
6	77.21%	80.36%	80.39%

**Table 7:** Confusion matrix of the proposed architecture.

		Predict		Accuracy
		Fire	No Fire	
Actual	Fire	4283	854	83.38%
	No Fire	638	2842	81.67%

**Table 8:** The test accuracy comparison for different bit-width of activation.

Integer Bits	Decimal Bits	Exit1 Accuracy	Exit2 Accuracy	Exit3 Accuracy
3	7	78.35%	82.16%	82.60%
3	6	78.31%	82.17%	82.57%
**3**	**5**	**78.29%**	**81.85%**	**81.94%**
3	4	75.37%	78.67%	78.97%

**Table 9:** The test accuracy comparison table for the bit-width of $\gamma^{\prime }$.

$\gamma^{\prime }$ Fixed Point Comparison Table				
Integer Bits	Decimal Bits	Exit1 Accuracy	Exit2 Accuracy	Exit3 Accuracy
3	11	78.32%	81.77%	81.92%
3	10	78.35%	82.02%	81.89%
3	9	78.29%	81.83%	81.85%
**3**	**8**	**78.26%**	**81.76%**	**81.88%**
3	7	59.72%	60.40%	60.76%

**Table 10:** The test accuracy comparison table for the bit-width of $\beta^{\prime }$.

$\beta^{\prime }$ Fixed Point Comparison Table				
Integer Bits	Decimal Bits	Exit1 Accuracy	Exit2 Accuracy	Exit3 Accuracy
3	7	78.26%	81.76%	81.92%
3	6	78.24%	81.85%	81.88%
3	5	78.18%	81.77%	81.84%
**3**	**4**	**78.16%**	**81.73%**	**81.82%**
3	3	77.47%	79.06%	79.04%

**Table 11:** The test accuracy comparison table for different bit-width of weight.

Comparison for Bit-Width of Weight				
Integer Bits	Decimal Bits	Exit1 Accuracy	Exit2 Accuracy	Exit3 Accuracy
2	9	78.08%	81.68%	81.76%
**2**	**8**	**78.06%**	**81.63%**	**81.73%**
2	7	76.12%	78.77%	79.25%

**Table 12:** The memory usage of each layer.

Store Data	Data Size (Height × Width × Channel × Bit-Width)	Total Size (bits)	Data Management
Input data	64 × 64 × 3 × 8	98,306	Sr1-1, Sr1-2
Psum of layer 1	64 × 64 × 1 × 19	77,824	Psum1, Psum2, Psum3, Psum4
Feature map of layer 1	32 × 32 × 8 × 8	65,536	rf1-1, rf1-2, rf1-3, rf1-4
Psum of layer 2	32 × 32 × 1 × 19	19,456	Psum1
Feature map of layer 2	16 × 16 × 8 × 8	16,384	Sr1-2
Psum of layer 3	16 × 16 × 1 × 19	4864	Psum1
Feature map of layer 3	8 × 8 × 16 × 8	8192	rf1-1
Psum of layer 4	8 × 8 × 1 × 19	1216	Psum1
Feature map of layer 4	4 × 4 × 32 × 8	4096	rf1-2

**Table 13:** Memory reduction ratio information.

Memory Type	Memory	Total Bits before the Fixed-Point	Total Bits after the Fixed-Point	Reduction Ratio
RAM	Sr1-1, Sr1-2	393,224	98,306	75%
Register	Psum1~4	131,072	77,824	40.625%
	rf1-1, rf1-2, rf1-3, rf1-4	262,144	65,536	75%
ROM	rom_c1c2, rom_c3, rom_c4	209,920	45,920	78.125%
	rom_exit1, rom_exit2, rom_exit3	145,408	31,808	78.125%
The sum of all bits		1,141,768	319,394	72.03%

**Table 14:** The test accuracy software to hardware in different stages.

Operation of Each Stage	Exit1 Test Accuracy	Exit2 Test Accuracy	Exit3 Test Accuracy
Build CNN model	79.02%	83.04%	83.11%
Weight quantization to 7-bit by DoReFaNet	78.35%	82.21%	82.69%
Convert activation to 8-bit	78.29%	81.85%	81.94%
Convert $\gamma^{\prime }$ in BN to 11 bits	78.26%	81.76%	81.88%
Convert $\beta^{\prime }$ in BN to 7 bits	78.16%	81.73%	81.82%
Verilog register transfer	78.02%	81.47%	81.49%

**Table 15:** Comparison with prior methods.

	[6] Computer Networks’21	[8] Sensors’22	[13] Forests’22	Proposed Work	
Architecture	Xception	DenseNet201+ EfficientB5	FT-ResNet50	2-D CNN	
Hardware information	NVIDIA Geforce RTX 2080ti	NVIDIA Geforce RTX 2080ti	NVIDIA Geforce RTX 2080ti	Raspberry pi3 Model B	40 nm ASIC
Inference time (s)	N/A	0.018	0.055	3.809	0.077 (Exit1) 0.105 (Exit3) @300 MHz
Dataset	The FLAME dataset	The FLAME dataset	The FLAME dataset	The FLAME dataset	
Input size	254 × 254	254 × 254	254 × 254	64 × 64	
Parameters	22.9 M	50.7 M	25.6 M	11.2 k	
Quantization method	No	No	No	DoReFaNet+Fixed-point	
Accuracy	76.23%	85.12%	79.48%	81.82%	81.49%
Power consumption	600 W	600 W	600 W	≈2.5 W	117 mW@300 MHz

## Data Availability

Data are contained within the article.
